# Distinct Plasma Proteomic Signatures Distinguish MASLD From Non‐Steatotic Individuals but Not From MetALD

**DOI:** 10.1111/liv.70873

**Published:** 2026-09-29

**Authors:** Niharika Jakhar, Tobias Seibel, Maria Mironova, Corinna Meeßen, Jan Clusmann, Yazhou Chen, Thriveni B. Raju, Paul‐Henry Koop, Juliette E. Pearce, Ivan G. Costa, Thomas Stiehl, Andreas Schuppert, Kai Markus Schneider, Yaron Rotman, Carolin V. Schneider

**Affiliations:** ^1^ Medical Clinic III, Gastroenterology, Metabolic Diseases and Intensive Care University Hospital RWTH Aachen Aachen Germany; ^2^ Liver Diseases Branch National Institute of Diabetes and Digestive and Kidney Diseases, National Institutes of Health Bethesda Maryland USA; ^3^ Division of Gastroenterology and Hepatology University of California San Diego San Diego California USA; ^4^ Else Kroener Fresenius Center for Digital Health, Faculty of Medicine Carl Gustav Carus TUD Dresden University of Technology Dresden Germany; ^5^ German Cancer Consortium (DKTK) Partner Site Dresden and German Cancer Research Center (DKFZ) Heidelberg Germany; ^6^ Department of Medicine I Faculty of Medicine and University Hospital Carl Gustav Carus, TUD Dresden University of Technology Dresden Germany; ^7^ Institute for Cell and Tumor Biology RWTH Aachen University, University Hospital Aachen Aachen Germany; ^8^ Institute for Computational Genomics University Hospital RWTH Aachen Aachen Germany; ^9^ Uniklinik RWTH Aachen, Institute for Computational Biomedicine & Disease Modeling RWTH Aachen University Aachen Germany; ^10^ Department for Science and Environment Roskilde University Roskilde Denmark; ^11^ Center for Computational Life Sciences RWTH Aachen University Aachen Germany; ^12^ Institute for Computational Biomedicine, JRC for Computational Biomedicine RWTH Aachen University, University Hospital Aachen Aachen Germany

**Keywords:** differential protein expression analysis, MASLD, pathway enrichment, proteomics, steatotic liver disease

## Abstract

**Background and Aims:**

Metabolic dysfunction associated steatotic liver disease (MASLD) is the most prevalent liver disease, yet accurate early detection remains challenging. A particular diagnostic obstacle is distinguishing MASLD from metabolic dysfunction and alcohol‐related liver disease (MetALD), currently defined using clinically informed moderate alcohol consumption thresholds without biological validation. This study aimed to identify and externally validate plasma proteomic signatures associated with MASLD and assess whether proteomic profiles support a molecular distinction between MASLD and MetALD as currently defined.

**Methods:**

We analysed plasma proteomic profiles using OLINK data from UK Biobank (UKB) participants with liver MRI. MASLD was defined as MRI‐PDFF ≥ 5% and metabolic dysfunction (*n* = 165), MetALD (*n* = 46) as MASLD with additional moderate alcohol intake and controls as PDFF < 5% (*n* = 741).

**Results:**

Compared to controls, participants with MASLD showed 17 upregulated and 3 downregulated proteins after Bonferroni correction (adjusted *p* < 0.05). Among upregulated proteins, leptin (LEP), fatty acid‐binding protein, liver (FABP1) and aminoacylase1 (ACY1) and among downregulated, insulin‐like growth factor‐binding protein 1 (IGFBP1) were externally validated in an independent cohort (clinicaltrials.gov NCT02520609, *n* = 47). Individuals with MetALD showed no significant proteomic distinction from MASLD. Although the small MetALD sample size may have limited detection of differences, direct differential expression analysis did not identify statistically significant differences between MASLD and MetALD under the current clinical thresholds.

**Conclusions:**

MASLD was associated with a reproducible plasma proteomic signature enriched for proteins involved in lipid metabolism, inflammation and hormonal regulation. We did not identify a clear proteomic distinction between MASLD and MetALD under the current alcohol thresholds. However, this finding should be interpreted considering limited power and potential alcohol‐exposure misclassification.

## Key Points

1. The study investigated circulating proteomic profiles of the UK Biobank participants associated with MASLD and MetALD.
2. MASLD and MetALD showed clear proteomic differences from non‐steatotic controls, but no clear differentiation of MASLD and MetALD.
3. External validation supports directional consistency for four overlapping proteins, supporting MASLD‐associated proteomic signals.

## Lay Summary

The study investigated circulating protein markers of MASLD and MetALD using UK Biobank participants with both liver MRI and plasma proteomics data. Participants were classified as healthy controls, MASLD, or MetALD based on liver fat, alcohol intake, cardiometabolic risk factors and clinical information.

The analysis identified several proteins that differed between MASLD or MetALD patients with controls, after accounting for age, sex, and BMI. However, MASLD and MetALD showed substantial overlap in their overall proteomics profiles, with no strong protein‐level differences between the two disease groups.

Overall, the findings suggest that plasma proteomics can help identify non‐invasive markers of steatotic liver disease, while larger studies may be needed to detect more subtle differences between MASLD and MetALD.

**Abbreviations**
ACY1: aminoacylase
ADH4: alcohol dehydrogenase
ALD: alcohol‐related liver disease
AUD: alcohol use disorder
CDT: carbohydrate‐deficient transferrin
CES1: carboxylesterase
CETP: cholesteryl ester transfer protein
CGA: glycoprotein hormones alpha polypeptide
EtG: ethyl glucuronide liver
FABP1: fatty acid‐binding protein
FDR: false discovery rate
FGF21: fibroblast growth factor
FLD: fatty liver disease
GGT: gamma‐glutamyl transpeptidase
GH1: growth hormone
GSEA: gene set enrichment analysis
GSTA1: glutathione S‐transferase A1
GSTA3: glutathione S‐transferase A3
IGFBP1: insulin‐like growth factor‐binding protein
IGFBP2: insulin‐like growth factor‐binding protein 2
LEP: leptin
MASLD: metabolic dysfunction associated steatotic liver disease
MetALD: metabolic dysfunction and alcohol‐related liver disease
MS: mass spectrometry
NHS: National Health Service
NPX: Normalized protein eXpression
PDFF: proton density fat fraction
PEA: proximity extension assay
PEth: phosphatidylethanol
SLD: steatotic liver disease
UKB: UK Biobank

## 1 Introduction

Steatotic Liver Disease (SLD) is a leading cause of liver‐related morbidity and mortality worldwide [1]. The traditional nomenclature for liver diseases was updated in 2024 based on a recent Delphi consensus to better categorize metabolic liver diseases. The previous fatty liver disease is now SLD, with MASLD and MetALD among its subclasses. The current clinical classification of MetALD relies primarily on self‐reported alcohol consumption, with proposed thresholds of 20–50 g/day for women and 30–60 g/day for men [2]. However, these thresholds are based on expert consensus rather than biological evidence. This is problematic for two reasons: first, self‐reported alcohol intake is prone to underreporting and social desirability bias; and second, it remains unclear whether individuals with moderate alcohol consumption and concurrent metabolic dysfunction truly represent a distinct pathophysiological entity, or merely a variation within the MASLD‐alcohol‐related liver disease (ALD) spectrum [3]. Indirect biomarkers such as carbohydrate‐deficient transferrin (CDT), gamma‐glutamyl transpeptidase (GGT) and cholesteryl ester transfer protein (CETP) offer enhanced diagnostic sensitivity for heavy drinking [3]. More recently, direct ethanol metabolites including urinary and hair ethyl glucuronide (EtG) and phosphatidylethanol (PEth) have shown high specificity and temporal resolution for assessing alcohol intake [4, 5]. Despite these advances, there remains a critical need for more versatile and disease‐relevant therapeutic targets that can accurately reflect underlying biological mechanisms and their metabolic consequences, particularly in patients with overlapping metabolic dysfunction and moderately increased alcohol consumption [3]. Proteomic profiling may be a promising approach to address this gap. Circulating proteins, which reflect complex physiological and pathophysiological processes, may serve as indicators of metabolic and alcohol‐related liver damage [6]. Plasma proteins, predominantly synthesized in the liver, are central to many critical physiological functions, including synthesizing albumin, fibrinogen, coagulation and complement proteins and numerous transport proteins that contribute to systemic homeostasis [7]. In the context of steatohepatitis, proteomic approaches have identified distinct serum protein signatures that correlate with key histological features, such as steatosis, inflammation, ballooning and fibrosis. For instance, Sanyal et al. demonstrated that a panel of 37 serum proteins accurately predicted the presence of these histological features highlighting the potential of proteomics as a non‐invasive ‘liquid biopsy’ tool [8]. Another study showed that mass spectrometry (MS)‐based proteomics can serve as a diagnostic and prognostic approach for ALD, potentially applicable to routine clinical practice [9]. A recent UK Biobank (UKB) study by Liu et al. used proteomic and metabolomic profiles to distinguish ALD from MASLD and then molecularly subtype MetALD into alcohol‐predominant and cardiometabolic‐predominant groups [10]. However, proteomic changes of imaging‐based MASLD and MetALD have not yet been explored. To analyse shared and distinct proteomic signatures of MASLD from MetALD compared to controls, our study uses proteomics data from UKB and an independent cohort. The primary aim of our study was to identify and validate circulating protein signatures associated with MASLD. The secondary aim was to evaluate whether individuals classified as MetALD, based solely on self‐reported alcohol intake thresholds, exhibit distinct proteomic profiles when compared to MASLD, to determine whether current classification criteria are accompanied by measurable proteomic differences.

## 2 Materials and Methods

### 2.1 Exploration Cohort

The UKB (https://www.ukbiobank.ac.uk/) is a large‐scale cohort recruited from the general UK population. It includes extensive longitudinal data, such as health records, biochemical analyses, and imaging data for approximately 500,000 participants aged 40–69 years, recruited between 2006 and 2010, with ongoing follow‐up through linkage to National Health Service (NHS) records and repeat assessments at selected time points, as illustrated in Figure 1.

**FIGURE 1:**
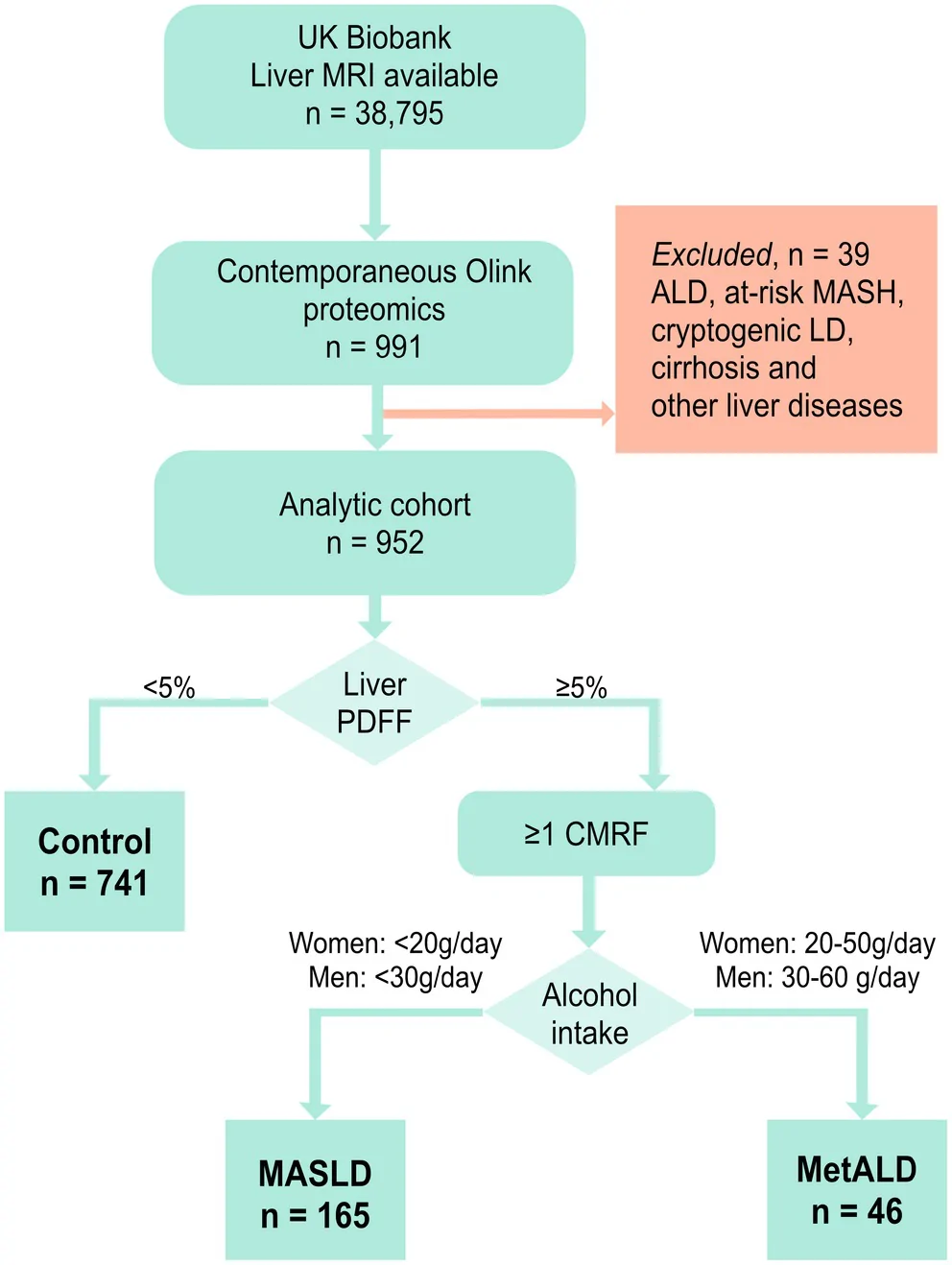
Flowchart of UKB cohort selection and study design: Participants with liver MRI were linked to proteomic measurements at the imaging visit. After exclusion of 39 participants meeting predefined criteria for ALD, at‐risk MASH, cryptogenic liver disease, cirrhosis or other liver disease, the final analytic cohort comprised 741 non‐steatotic controls, 165 participants with MASLD and 46 with MetALD. Classification was based on MRI‐derived PDFF, cardiometabolic risk factors, alcohol intake and the exclusion criteria detailed in the Methods and Appendix S1.

#### 2.1.1 Determination of Liver Fat Content

Liver fat content was estimated using MRI‐based proton density fat fraction (PDFF), which provides a non‐invasive proxy for the proportion of fat within the liver and serves as a diagnostic marker for steatosis at the second follow up of the UKB cohort (*n* = 38,795) [11].

#### 2.1.2 Connection of MRI With Proteomics Data at UKB Instance 2

Blood samples from a randomly selected subset of participants were analysed using OLINK's proximity extension assay (PEA) platform, which provides Normalized protein eXpression (NPX) values (measured on a log_2_ scale) as an indicator of protein concentration relative to other samples in the cohort [12]. For this study, we focused on the subset of individuals with available proteomics data at the time of MRI (Figure 1). Proteomics measurements at Instance 2 correspond to the imaging visit (UKB Field 53–2). Nine hundred and ninety‐one of the participants with liver MRI have proteomics data available at the second instance.

#### 2.1.3 Cardiometabolic Risk Stratification [2]

To characterize the metabolic profile of the cohort, individuals were stratified based on established cardiometabolic risk factors, aligning with MASLD diagnostic criteria. Five metabolic conditions were defined in alignment with recent consensus statements [2]:

1. Obesity or Central Adiposity: BMI ≥ 25 kg/m^2^ (for ‘Asian’ BMI ≥ 23 kg/m^2^) or waist circumference ≥ 80 cm (women) or ≥ 94 cm (men).
2. Impaired Glucose Metabolism or Diabetes: Fasting glucose ≥ 100 mg/dL and fasting time ≥ 8 h, HbA1c ≥ 5.7%, diagnosis of type 2 diabetes or use of antidiabetic medication.
3. Hypertension: Systolic BP ≥ 130 mmHg, diastolic BP ≥ 85 mmHg or use of antihypertensive medication.
4. Hypertriglyceridemia: Triglycerides ≥ 1.7 mmol/L or 150 mg/dL or use of lipid‐lowering therapy.
5. Low HDL Cholesterol: HDL ≤ 50 mg/dL (women) or ≤ 40 mg/dL (men), or use of lipid‐lowering therapy.

#### 2.1.4 Alcohol Consumption Data Processing

Alcohol consumption was assessed using responses to a standardized questionnaire capturing frequency and quantity of intake across various alcoholic beverages, beer/cider, red wine, white wine/champagne, spirits, fortified wine and other beverages. Weekly consumption was captured via fields 1568‐2.0 to 5364‐2.0, and monthly consumption via fields 4407‐2.0 to 4462‐2.0 and combined to a daily consumption [13] (Appendix S1). To facilitate comparison with the recent UK Biobank analysis by Liu et al. [10], we report the clinical alcohol‐category boundaries in both daily and weekly units.

#### 2.1.5 Classification and Grouping

Participants meeting eligibility criteria designed to ensure accurate phenotyping were stratified by daily alcohol consumption and further classified into:

MASLD: Participants with a PDFF value ≥ 5%, one or more cardiometabolic risk factors, but no elevated alcohol consumption (< 20 g/day for women and < 30 g/day for men).

MetALD: Participants with PDFF ≥ 5%, one or more cardiometabolic risk factors, and alcohol intake within the risk thresholds (20–50 g/day [140–350 g/week] for women and 30–60 g/day [210–420 g/week] for men).

Control: Participants with a PDFF lower than 5%, with no evidence of steatosis.

We selected participants with available proteomics data. To avoid proteomic differences that relate to cirrhosis and advanced fibrosis (which may differ between MASLD and MetALD), we excluded patients with cirrhosis and advanced fibrosis defined as patients with cT1 ≥ 875 ms (corrected T1 refers to the longitudinal relaxation time in MRI, correlated with fibrosis), as this points towards advanced fibrosis (at‐risk MASH) as defined by the new MASLD guideline [14] as well as patients with ALD (*n* = 26), see Appendix S1.

The final included participants were MASLD (*n* = 165), MetALD (*n* = 46) and Controls (*n* = 741).

Detailed description of alcohol consumption calculations and definitions of other excluded diseases are presented in Appendix S1.

#### 2.1.6 Demographic and Health Variables

In addition to imaging‐derived measures, we incorporated participant‐level data on age, sex, waist circumference and BMI (Table 1). Smoking status was also reported, based on self‐reported questionnaire data.

**TABLE 1:** Baseline characteristics of the discovery cohort stratified in three groups: Participants were grouped as controls, MASLD and MetALD according to the study phenotype definitions.

	Control (*n* = 741)	MASLD (*n* = 165)	MetALD (*n* = 46)	*p*‐Value (all)
Variable				
Age at recruitment (years)	49.0 (44.0–54.0)	50.0 (46.0–55.0)	54.0 (45.0–57.0)	5.449 × 10^−2^
Body mass index (BMI)	24.7 (22.6–27.2)	28.4 (26.3–31.2)	28.9 (26.6–30.9)	1.707 × 10^−32^
Waist circumference (cm)	81.0 (74.0–91.0)	95.0 (86.0–102.0)	96.0 (90.0–103.0)	2.644 × 10^−34^
Weight (kg)	70.9 (62.6–81.5)	84.0 (73.4–94.2)	87.7 (77.8–96.3)	3.298 × 10^−27^
Male	39.1%	63.6%	67.4%	5.116 × 10^−10^
White	90.4%	90.9%	91.3%	9.654 × 10^−01^
Lifestyle				
Smoking status (current)	30.5%	30.9%	43.5%	1.821 × 10^−1^
Alcohol consumption (g/d)	11.0 (3.5–20.7)	9.7 (2.0–17.1)	37.5 (32.2–40.0)	6.236 × 10^−23^
Liver fat content (%)	2.40 (1.90–3.20)	8.80 (6.60–13.40)	8.15 (6.33–12.82)	1.072 × 10^−107^

*Note:* Continuous variables (age, body mass index (BMI), weight (in kg) and waist circumference (in cm)) are presented as median with interquartile range. Overall group differences across the three groups were computed using the Kruskal‐Wallis test. Categorical variables (sex, ethnic background and smoking status are presented as percentages). Overall differences in categorical variables are assessed using chi‐square tests, and pairwise comparisons versus controls were assessed using chi‐square or Fisher's exact tests, as appropriate. *p* values indicate the level of statistical evidence for differences between groups.

### 2.2 Statistical Analysis

Continuous variables, including age and BMI, are displayed as median (IQR) and were compared across the three groups (Control, MASLD and MetALD) using the Kruskal–Wallis test. Categorical variables, such as sex, smoking history and ethnicity, are displayed and were tested for significant differences across groups using the Chi‐square test. Alpha‐level was defined at *p* < 0.05. All statistical analyses were performed using Python version 3.11.5. The following Python packages were used for data processing and statistical testing:

Pandas version 2.1.4 for data manipulation and tabulation numpy version 1.26.4 for numerical computations. Scipy version 1.12.0 for conducting non‐parametric statistical tests, including the Kruskal–Wallis. Analyses were carried out in a reproducible programming environment using Python scripts.

#### 2.2.1 Missingness Assessment

Protein‐level missingness was assessed for each disease comparison. For every protein, the number and proportion of missing values were calculated overall (Table S1), as missingness was very low.

Regression models were fitted using available complete cases for each protein, meaning that participants were included in each protein model only if they had non‐missing values for the protein, disease status and all covariates included in the model. Missingness metrics were added to the protein‐level results tables (Table S1) to allow assessment of potential bias. Group‐specific missingness was assessed internally but was not presented as reporting could violate the UKB small‐number policy.

#### 2.2.2 Differential Expression Analysis

We performed differential protein expression analysis between control and disease groups. Protein expression values were analysed on the log_2_ Olink NPX scale (Figure 2). For this analysis, the dataset was first split into separate control and disease groups based on predefined labels as mentioned above. For each protein, a multivariable linear regression model with protein expression as the outcome and disease status as the main exposure was fitted. Disease status was coded as a binary variable for each comparison. The primary adjusted model included age, sex, body mass index (BMI) as prespecified covariates: protein abundance ~ disease status + age + sex + BMI. These protein‐based adjusted models were fitted using complete cases for the protein measurements and covariates included in the model. Participants with missing covariate data were excluded from the adjusted analysis. The final adjusted analysis therefore included 741 controls, 164 participants with MASLD and 46 participants with MetALD because one MASLD participant had missing covariate data.

**FIGURE 2:**
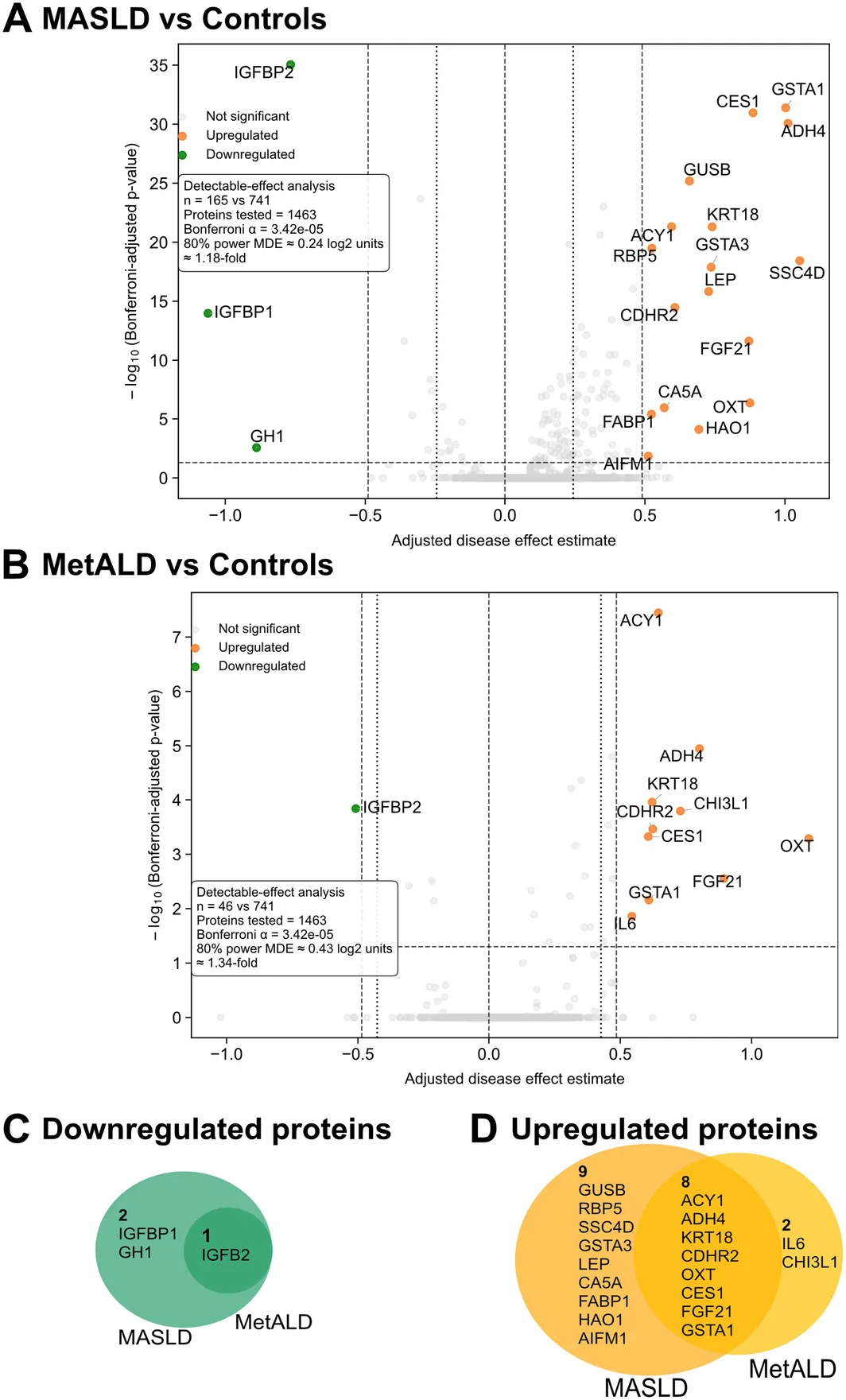
Proteomic landscape of MASLD and MetALD. (A) Volcano plot for MASLD versus controls (*n* = 1 vs. 741) and (B) MetALD versus controls (*n* = 46 vs. 741) from protein‐wise linear models adjusted for age, sex and BMI. The x‐axis shows the adjusted disease‐status coefficient and the *y*‐axis −log10(Bonferroni‐adjusted *p*‐value); highlighted proteins meet the prespecified statistical and effect‐size criteria. Reference lines indicate the significance/effect‐size thresholds and the detectable‐effect values summarized in the panel annotations. (C) Overlap of downregulated proteins and (D) overlap of upregulated proteins identified in the two case–control comparisons.

The regression coefficient for disease status was used as the adjusted disease effect estimate. A positive adjusted beta indicated higher protein abundance in the disease group compared to the reference group, whereas a negative adjusted beta indicated lower protein abundance in the disease group. For each protein, we extracted the adjusted disease effect estimate, 95% confidence interval, nominal *p*‐value and model sample size. *p*‐Values from the adjusted disease‐status coefficient were corrected for multiple testing using Bonferroni correction across all tested proteins. Proteins were classified as differentially abundant if they met both the Bonferroni‐adjusted significance threshold and the prespecified absolute effect‐size threshold. Because protein values were analysed on a log_2_ scale, the effect‐size threshold was defined as an absolute adjusted beta greater than log_2_(1.5), corresponding approximately to a 1.5‐fold difference on the original abundance scale (Tables S2 and S3).

Volcano plots were generated using the adjusted disease effect estimate on the *x*‐axis and the negative log_10_ Bonferroni‐adjusted *p*‐value on the *y*‐axis. Proteins passing both the adjusted significance and effect‐size thresholds were highlighted as upregulated or downregulated.

##### 2.2.2.1 Minimum Detectable Effect Analysis

For the MASLD versus MetALD comparison, a minimum detectable effect analysis was done to assess whether the absence of significant proteins could reflect limited statistical power. This analysis was motivated by the smaller MetALD sample size and the strict Bonferroni correction applied across all tested proteins.

For each comparison, the Bonferroni‐corrected alpha threshold was calculated as 0.05 divided by the number of proteins tested. We then estimated the minimum detectable standardized effect size required to achieve 80% power using the observed group sizes and the Bonferroni‐corrected alpha threshold. To aid interpretation on the protein scale, the standardized detectable effect was converted into an approximate detectable log_2_ effect using the median protein standard deviation across tested proteins. This detectable log_2_ effect was then converted to an approximate fold change as 2^ß^.

The detectable‐effect analysis was used to contextualize non‐significant findings, particularly for the MASLD versus MetALD comparison. This analysis was not used to define differential abundance, but to indicate the approximate magnitude of protein differences that the study was powered to detect under the applied multiple‐testing threshold.

##### 2.2.2.2 Alcohol Exposure Sensitivity Analysis

Because alcohol intake was based on self‐report, we performed exploratory analyses to assess whether alcohol‐consumption categories were associated with broad proteomic structure. Alcohol intake was categorized as low (< 20 g/day), moderate (20–30 g/day) or high (> 30 g/day) (reference: Appendix S1). Principal component analysis (PCA) was performed using proteomic profiles from participants in the MASLD and MetALD comparison, and samples were visualized according to alcohol‐consumption category. As a complementary nonlinear visualization, t‐distributed stochastic neighbour embedding (t‐SNE) was applied to the same participant‐by‐protein matrix and projected into two dimensions; the embedding was used only for exploratory visualization and not for hypothesis testing or disease classification. Because the clinical MetALD alcohol thresholds are sex‐specific, the PCA was also repeated separately in women and men.

#### 2.2.3 Pathway Analysis

To explore biological processes represented among the differentially abundant proteins, we performed over‐representation analysis using Enrichr, optimized for a small target list, for the 17 upregulated proteins in MASLD [15]. Analysis was performed against the default background set of proteins for comparison. For upregulated proteins among MASLD versus Control, gene names were queried in the STRING database to explore the functional significance of MASLD associated proteins [16]. Gene set enrichment analysis for the top 10 pathways with significantly altered proteins was performed against KEGG 2021 Human database [17]. A gene‐concept network was constructed to illustrate the relationships between enriched pathways and their associated genes using EnrichR‐KG [18] (Appendix S1).

### 2.3 External Validation

The validation cohort was previously described in Velenosi et al. [19]. Briefly, in a prospective study of metabolism (clinicaltrials.gov NCT02520609), 37 adult individuals with suspected non‐alcoholic fatty liver disease (cases) and 10 adult healthy controls were enrolled. Steatosis was confirmed by imaging, and significant alcohol consumption (> 30 g/d for men or > 20 g/d for women or binge drinking) was excluded. While the study was conducted prior to the revised nomenclature of the disease, we confirmed that all cases met the current MASLD criteria [2]. Healthy controls had normal BMI, ALT and no evidence of steatosis on ultrasound. Plasma samples were collected at 8AM after a 14‐h fast and stored at −80°C until further analysis. 1285 unique proteins were analysed using SomaLogic assay v3.2 (SomaLogic, Boulder, CO). Protein concentrations were expressed in relative fluorescence units and log‐transformed for analysis.

For this analysis, we set out to study all proteins that were significantly enriched in MASLD participants in our UKB analyses. All 20 significantly different proteins of the MASLD vs. control comparison in UKB were selected (Figure 3), of which 8 were available on both platforms.

**FIGURE 3:**
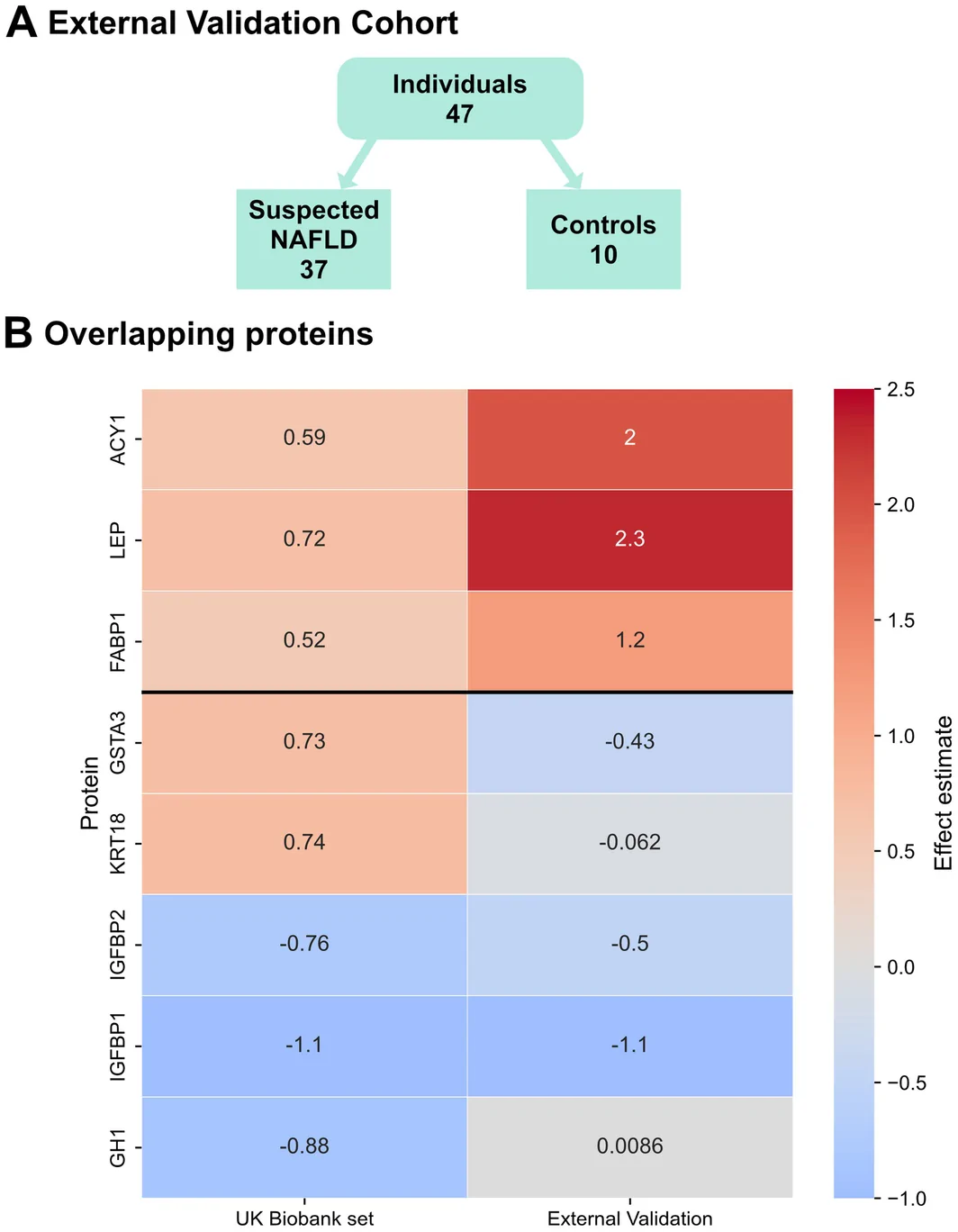
External validation of the MASLD‐associated proteomic signature. (A) Composition of the independent validation cohort (37 cases meeting current MASLD criteria and 10 healthy controls; total *n* = 47). (B) Heat map of the eight UKB discovery proteins measured on both proteomic platforms. Cell values show the cohort‐specific effect estimate displayed in the figure for MASLD versus controls; positive values indicate higher and negative values lower abundance in MASLD. LEP, FABP1, ACY1 and IGFBP1 showed significant changes in the same direction in the external cohort after BH‐FDR correction across the eight overlapping proteins. Because UKB and validation samples were measured on different platforms, absolute effect magnitudes should be interpreted within cohort rather than compared directly across platforms.

All data were normalized, calibrated and underwent a quality control check. Cohorts were processed independently, and no cross‐cohort batch correction or joint normalization was performed. For the external cohort, nominal significance was assessed with a two‐sided exact Wilcoxon rank‐sum (Mann–Whitney *U*) test. The eight UKB discovery proteins measurable on both platforms constituted a single external‐validation multiple‐testing family; Benjamini‐Hochberg FDR correction was therefore applied jointly across these eight nominal *p*‐values (*m* = 8), irrespective of direction of regulation, with *q* ≤ 0.05 considered significant. For external effect‐size reporting, the geometric fold change was calculated as 2^(mean log2 MASLD−mean log2 control)^, and Hedges' g with 95% confidence intervals was calculated on the log_2_‐transformed values.

#### 2.3.1 Compliance With Ethical Standards

All research was conducted in accordance with the Ethical Principles for Medical Research Involving Human Subjects outlined in the 2013 Declaration of Helsinki and the 2018 Declaration of Istanbul. This study used individual‐level data from the UK Biobank under approved application number 71300. The UK Biobank protocol was approved by the North West Multi‐centre Research Ethics Committee, UK (REC reference 11/NW/0382), and written informed consent was obtained from all participants at the time of enrolment. Summary‐level results from a previously conducted NIH‐funded clinical trial (ClinicalTrials.gov Identifier: NCT02520609) were used for external validation.

## 3 Results

### 3.1 Baseline Differences of MASLD, MetALD and Non‐Steatotic Individuals

Characteristics of participants with proteomic measurements and liver MRI included in this study, are summarized in Table 1. The 165 participants with MASLD and the 46 with MetALD had higher BMI, body weight, waist circumference and liver fat content than 741 non‐steatotic controls, consistent with more advanced hepatic steatosis. A higher proportion of males was observed in the MASLD and MetALD groups. However, there were no statistically significant differences in ethnic composition and smoking status across groups (Table 1).

#### 3.1.1 Protein Missingness

Protein‐level missingness was assessed before model fitting. Among the top MASLD discovery proteins, total missingness was low, ranging from 0% to 3.97% across proteins (Table S1). Accordingly, protein‐wise regression models were fitted using available complete cases for each protein and the included covariates. Model sample sizes ranged from 869 to 905 participants (MASLD, controls), reflecting the small amount of protein‐specific missingness (Table S1).

### 3.2 Differential Protein Abundance in MASLD Versus Controls

First, we focused on the differences between MASLD and controls. Here, we performed protein‐wise multivariable linear regression to assess differential protein abundance after adjustment for age, sex and BMI (Figure 2A). We identified 20 differentially abundant proteins in MASLD compared to controls (after Bonferroni correction and application of the effect‐size threshold of |β| > log_2_(1.5)). Those included the upregulated proteins alcohol dehydrogenase 4 (ADH4), carboxylesterase 1 (CES1), leptin (LEP), aminoacylase 1 (ACY1), fatty acid‐binding protein 1 (FABP1) and glutathione S‐transferase A1 (GSTA1) among others, complete list provided in Figure 2A and Table S2. We further identified three downregulated proteins in MASLD: insulin‐like growth factor‐binding protein 1 (IGFBP1), insulin‐like growth factor‐binding protein 2 (IGFBP2) and growth hormone 1 (GH1) (Table S3).

#### 3.2.1 External Validation of Differentially Abundant Proteins

We evaluated the robustness of the identified proteins to differentiate MASLD from controls in an independent validation cohort [19]. As proteomic analysis in the validation cohort utilized a different platform than the discovery cohort (SomaLogic rather than OLINK), analysis was limited to proteins present on both platforms. Among the 17 upregulated and 3 downregulated proteins in the UKB dataset, 8 were available in the validation cohort and were corrected jointly for multiple testing using Benjamini‐Hochberg FDR. Four proteins were significant in the same direction as in UKB: LEP (Hedges' *g* = 2.06, 95% CI 1.14–2.98; *q* = 0.0071), ACY1 (*g* = 1.85, 95% CI 0.95–2.74; *q* = 1.03e‐05), FABP1 (*g* = 1.29, 95% CI 0.44–2.14; *q* = 0.0148) and IGFBP1 (*g* = −1.21, 95% CI −2.06 to −0.37; *q* = 0.0364) (Figure 3, Table S4).

#### 3.2.2 Functional Characterization of MASLD‐Associated Proteins

A protein–protein interaction network of the upregulated proteins found in MASLD versus controls highlighted key such as LEP, FABP1, ACY1 and CES1, all of which are functionally linked to metabolic regulation, lipid handling and hormone signalling [20, 21, 22, 23]. Proteins such as GSTA1, glutathione S‐transferase A3 (GSTA3) and ADH4 clustered, suggesting a coordinated stress response (Figure 4A). The connectivity of these proteins highlights their potential cooperative roles in the metabolic reprogramming characteristic of MASLD (Figure 4A). Gene‐concept network analysis further supported these findings (Figure 4B), revealing that MASLD associated proteins were enriched in key hepatic pathways, particularly those involving glutathione metabolism, xenobiotic metabolism via cytochrome P450, and drug metabolism. Notably, proteins such as GSTA1, GSTA3 and ADH4 demonstrated high centrality within the metabolic network. Among the most significantly enriched terms and pathways were decreased liver triglyceride level, abnormal lipid level, abnormal hepatocyte physiology, drug metabolism, glutathione metabolism and cytochrome P450‐mediated xenobiotic metabolism. The latter pathways are closely linked to hepatic detoxification and redox homeostasis [24]. Additional enrichment was observed in pathways related to carcinogenesis, including chemical carcinogenesis, pathways in cancer, and HCC. Furthermore, involvement of pathways such as fluid shear stress and atherosclerosis and nitrogen metabolism point to systemic metabolic dysfunction with cardiovascular and renal implications. As these analyses are cross‐sectional, the observed enrichment does not establish malignant transformation or predict future HCC risk, which would require longitudinal follow‐up and independent validation (Figure 4B).

**FIGURE 4:**
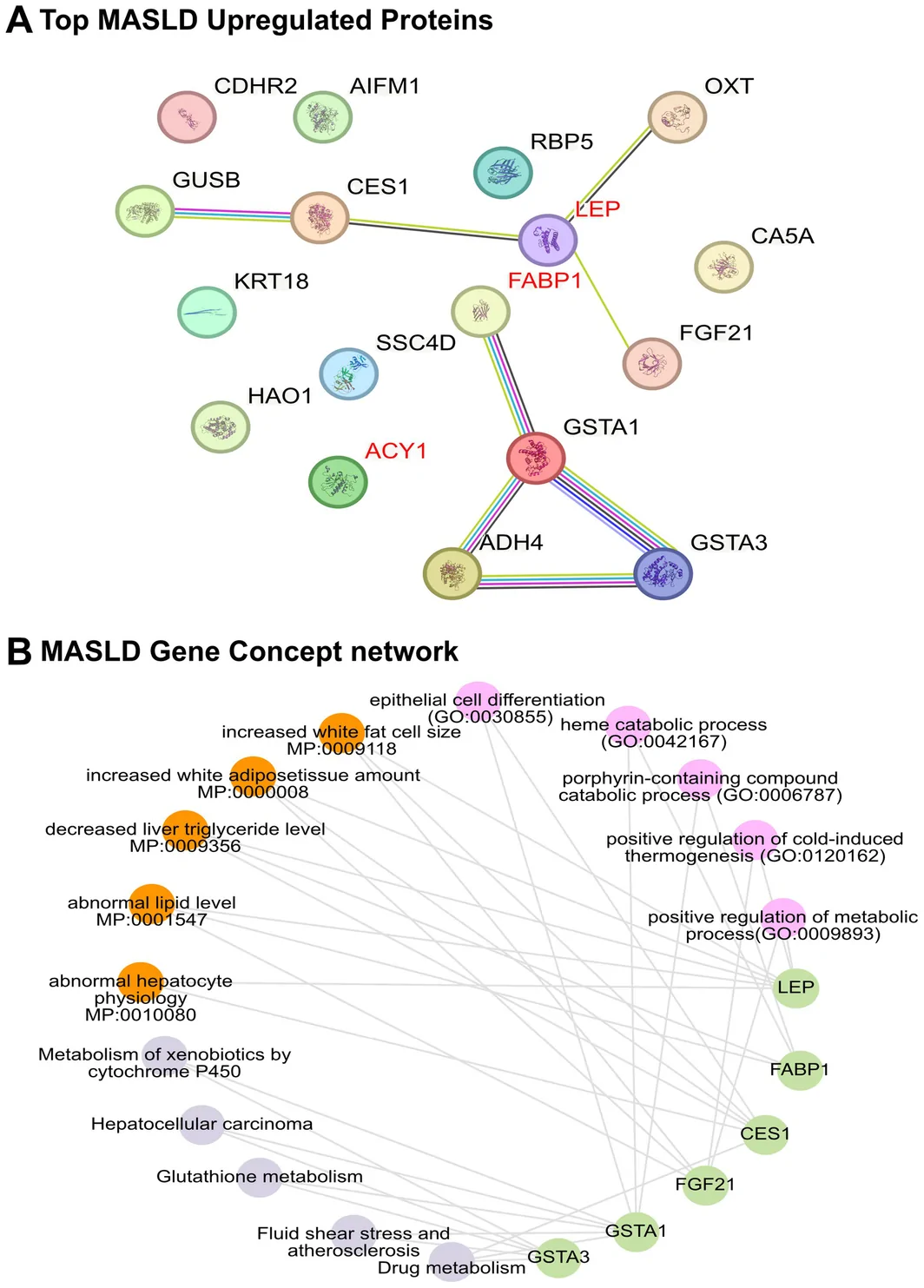
Functional characterization of proteins upregulated in MASLD. (A) STRING protein–protein interaction network for the 17 proteins upregulated in MASLD versus controls. Nodes represent proteins and edges represent STRING‐supported functional associations; the three proteins reproduced in the external cohort (ACY1, LEP and FABP1) are highlighted in red. (B) Gene‐concept network linking MASLD‐associated proteins to selected enriched biological pathways and phenotype terms. Protein nodes are connected to terms in which they are annotated. The network is intended as an exploratory visualization of functional overlap and does not imply causal relationships or disease progression.

### 3.3 Distinct Plasma Proteomic Signatures Distinguish MetALD From Non‐Steatotic Individuals but Not From MASLD

In the MetALD versus control comparison, 11 proteins were significantly differentially abundant after multivariable adjustment and Bonferroni correction, including 10 upregulated and 1 downregulated protein. Notably, IGFBP2 was also found to be downregulated in MetALD vs Controls. Among the upregulated proteins were ACY1, ADH4, KRT18, CHI3L1, CDHR2, CES1, OXT, FGF21, GSTA1 and IL6 (Figure 2B).

We then directly compared proteomic profiles of the two disease groups MASLD and MetALD to assess whether they showed distinct protein signatures. No proteins met the criteria for differential abundance after multivariable adjustment, Bonferroni correction and effect‐size filtering (Figure S1).

#### 3.3.1 Minimum Detectable Effect Analysis

Because the direct MASLD versus MetALD comparison had a modest MetALD sample size, we performed a minimum detectable effect analysis to contextualize the absence of significant proteins. Assuming 80% power and a two‐sided Bonferroni‐adjusted alpha of 0.05/1463, the minimum detectable standardized effect was *d* = 0.431 for MASLD versus Control, *d* = 0.762 for MetALD versus Control and *d* = 0.849 for the direct MASLD versus MetALD comparison. These values represent minimum detectable standardized effects under the stated assumptions, not observed effect sizes (Table S5). Cohen's *d* was converted to an approximated log_2_ NPX difference by multiplying *d* by the median standard deviation of log_2_ NPX values across the proteins analysed in each comparison. Approximate fold‐change was then calculated as 2^β^. This corresponded to approximate minimum detectable differences of 0.244, 0.427 and 0.460 log_2_ NPX units for MASLD versus controls, MetALD versus controls, and MASLD versus MetALD, respectively. Equivalent to approximately 1.184‐, 1.344‐ and 1.375‐fold differences (Table S5). These results indicate that the direct MASLD versus MetALD comparison was powered primarily to detect relatively large protein differences. Therefore, the absence of Bonferroni‐significant proteins should be represented as a lack of evidence for large, robust proteomic differences between MASLD and MetALD, rather than evidence excluding smaller differences.

#### 3.3.2 Exploratory Alcohol‐Consumption Analyses

To assess whether self‐reported alcohol consumption was associated with broad proteomic structure, we performed exploratory PCA and t‐SNE analyses in the MASLD and MetALD groups. Alcohol intake was categorized as low (< 20 g/day, *n* = 138), moderate (20–30 g/day, *n* = 32) and high (> 30 g/day, *n* = 41; Figure 5A). The first two principal components explained 18.0% and 7.5% of the variance, respectively. Participants showed substantial overlap across low, moderate and high alcohol‐intake groups, with no clear separation between MASLD and MetALD in the first two principal components; the complementary t‐SNE visualization likewise showed broad overlap without clear clustering by alcohol‐consumption category (Figure 5B). These exploratory findings were consistent with the adjusted MASLD‐versus‐MetALD differential abundance analysis, which did not identify Bonferroni‐significant proteins (Figure S1). Because both PCA and t‐SNE are unsupervised visualizations and alcohol intake was based on self‐reported questionnaires, these analyses were interpreted as exploratory. As the MetALD alcohol thresholds are sex‐specific, we additionally examined PCA separately in women and men; both sex‐stratified analyses showed substantial overlap across alcohol‐intake categories (Figure 6).

**FIGURE 5:**
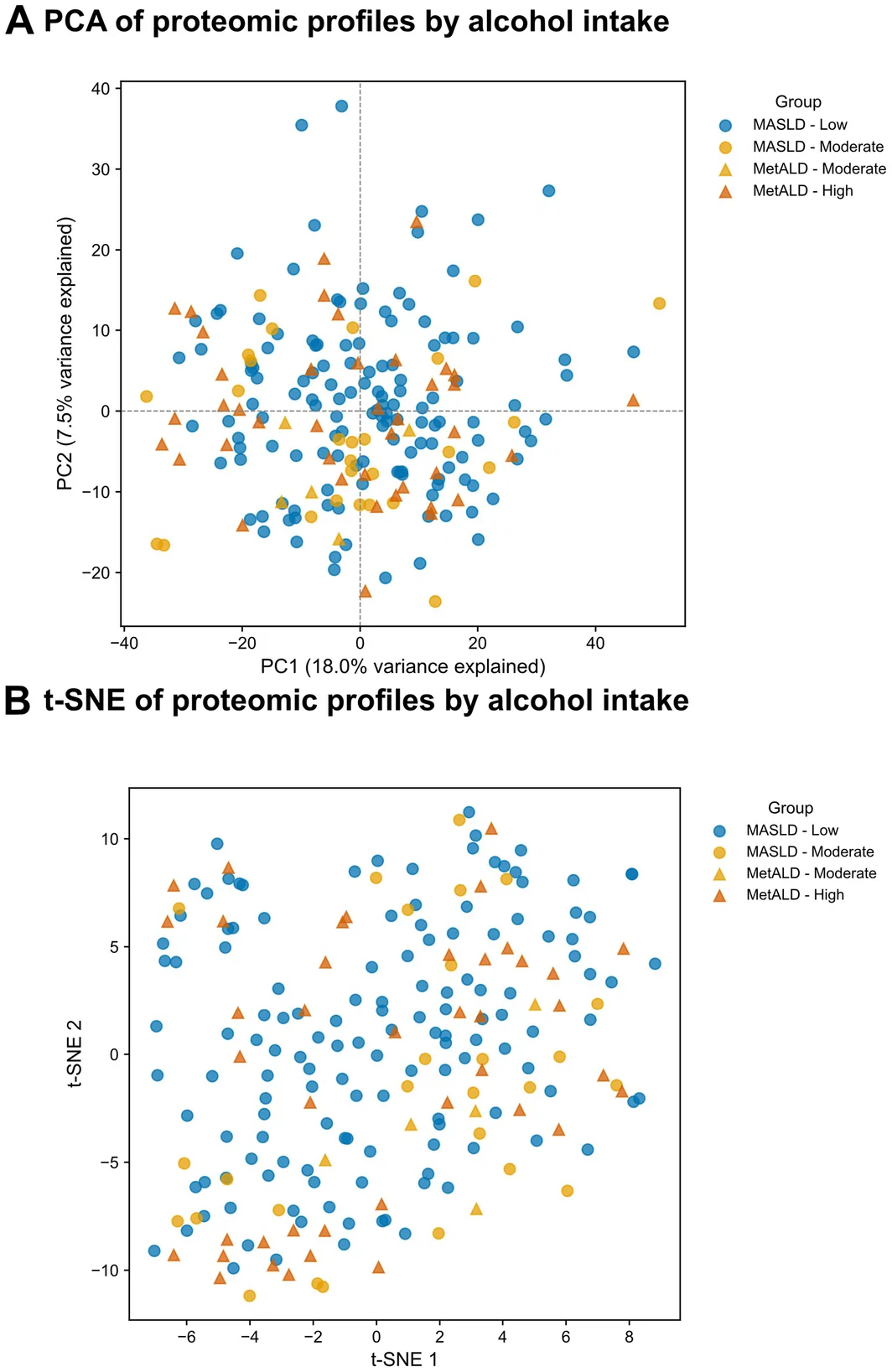
Exploratory principal component analysis (PCA) of global plasma proteomic profiles by alcohol exposure‐related categories. (A) PCA and (B) t‐distributed stochastic neighbour embedding (t‐SNE) of proteomic profiles of all 211 participants with MASLD or MetALD, grouped by self‐reported alcohol intake: Low < 20 g/day (*n* = 138: MASLD; *n* = 0: MetALD), moderate 20–30 g/day (*n* = 27: MASLD; *n* = 5: MetALD) and high > 30 g/day (*n* = 0 MASLD; *n* = 41: MetALD). PC1 and PC2 explain 18.0% and 7.5% of variance, respectively (25.5% combined). The broad overlap among groups indicates no clear global separation in the first two principal components.

**FIGURE 6:**
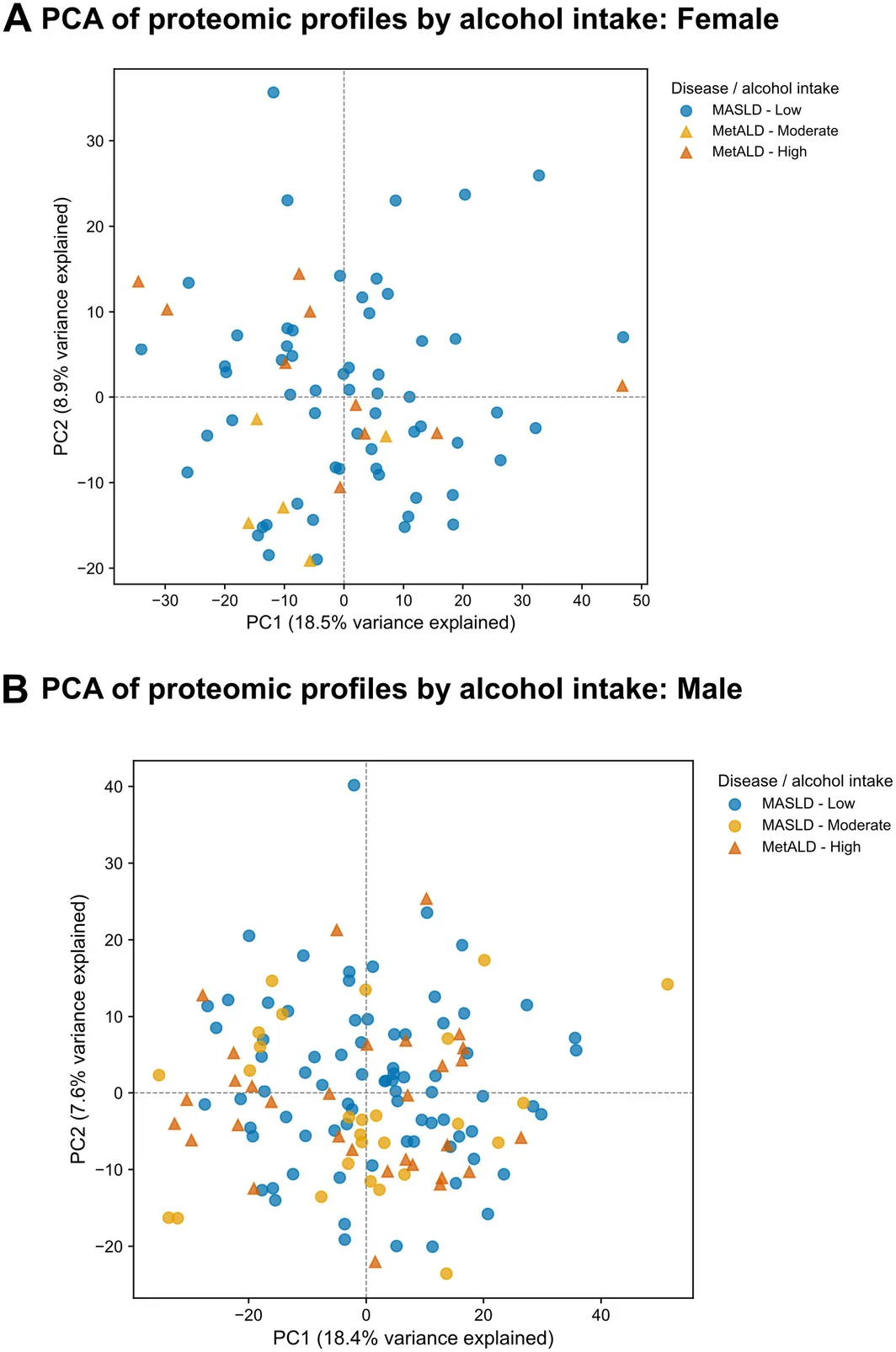
Exploratory PCA of proteomic profiles by SLD subgroup, alcohol and sex in (A) Female (*n* = 75) with MASLD and MetALD, stratified by alcohol intake: Low < 20 g/day (*n* = 60: MASLD; *n* = 0: MetALD), moderate 20–30 g/day (*n* = 0: MASLD, *n* = 5: MetALD), high > 30 g/day (*n* = 0: MASLD; *n* = 10: MetALD) and (B) Male (*n* = 136) with MASLD and MetALD, stratified by alcohol intake: Low < 20 g/day (*n* = 27: MASLD; *n* = 0: MetALD), moderate 20–30 g/day (*n* = 78: MASLD, *n* = 0: MetALD), high > 30 g/day (*n* = 0: MASLD; *n* = 31: MetALD). The broad overlap among groups indicates no clear global separation in the first two principal components.

## 4 Discussion

This study explored distinct proteomic profiles in SLD subtypes. Using same day liver MRI and plasma proteomics from UK Biobank, complemented by an independent validation cohort, we characterized the plasma proteomic landscape of MRI‐defined MASLD and assessed whether the current self‐reported alcohol‐based definition of MetALD is accompanied by a distinct molecular phenotype. First, we identified 17 upregulated and 3 downregulated proteins in MASLD compared to controls. Among the most strongly upregulated proteins in MASLD was GSTA1, an alpha‐class glutathione S‐transferase involved in antioxidant defence and detoxification [24]. Experimental studies have shown that GSTA1 interacts with FABP1 and facilitates its degradation, thereby reducing fatty‐acid uptake and intracellular triglyceride accumulation [22]. In hepatocytes and high‐fat‐diet‐fed mice, GSTA1 overexpression attenuated steatosis, while pharmacological upregulation of GSTA1 similarly reduced hepatic lipid accumulation, suggesting that GSTA1 may represent a potential therapeutic target in MASLD [22].

The second most strongly upregulated protein was ADH4. ADH4, which encodes Class II π‐alcohol dehydrogenase, is involved in ethanol metabolism and catalyses the oxidation of retinol to retinal, linking alcohol metabolism with retinoid homeostasis [25]. More recently, patients with MASLD were shown to have reduced ADH activity and elevated fasting blood ethanol concentrations despite the absence of alcohol consumption; experimental studies further implicated TNFα‐ and JNK‐dependent mechanisms in this reduction in ADH activity [26].

Another interesting finding was the upregulation of fibroblast growth factor 21 (FGF21). FGF21 is known to be involved in fibrogenesis and metabolic regulation, especially in lipid homeostasis and insulin sensitivity, making it a potential therapeutic target in MASLD [27]. FGF21 based medications are currently being explored as a novel therapeutic target for MASLD [28]. Since FGF21 is secreted from the liver into the plasma, our result supports the development of new treatments using FGF21 agonist mechanisms.

In our external validation cohort, we confirmed the differential expression of the upregulated ACY1, leptin and FABP1, further supporting their involvement in MASLD pathogenesis. ACY1 is an aminoacylase involved in amino‐acid metabolism through the hydrolysis of N‐acetylated amino acids [29]. Experimental studies have demonstrated that circulating ACY1 modulates N‐acetylated and free amino‐acid levels and influences insulin and glucose homeostasis in vivo, providing a potential link between ACY1 and systemic metabolic dysfunction [29]. More recently, a large prospective proteomic study published in 2026 identified ACY1 as one of five plasma proteins that predicted incident MASLD with predictive information detectable up to 16 years before clinical diagnosis [20]. Together with our independent validation of increased ACY1 in MASLD, these findings support ACY1 as a reproducible marker of hepatic steatosis.

Moreover, we could reproduce Leptin, an adipocyte‐derived hormone, that plays a critical role in lipid metabolism, inflammation and fibrosis [23]. It has been implicated in oxidative stress and Kupffer cell activation, contributing to the progression of liver disease [30]. Similarly, FABP1 was validated and it is essential for intracellular fatty acid transport and modulates lipid homeostasis and inflammation [22]. Increased hepatic FABP1 expression has been associated with exacerbation of steatosis and inflammatory responses, reinforcing its role in MASLD [22].

A second aim of the study was to evaluate whether individuals classified as MetALD, defined by moderate alcohol intake in the presence of metabolic dysfunction, exhibit proteomic features distinct from MASLD. Our results revealed a high degree of molecular overlap between the two groups (Figure 2C,D). Strikingly, IGFBP2 was downregulated in both MASLD and MetALD compared to controls, while IGFBP1 was among the validated proteins for MASLD versus controls. This was also validated in external validation. This is in line with previous studies that suggested a reduction of IGF, a key anabolic hormone, in subjects with advanced liver fibrosis [31]. IGFBP2 acts as an inhibitor in the epidermal growth factor receptor (EGFR)‐signal transducer and activator of transcription factor 3 (STAT3) pathway [32]. IGFBP2, bound to EGFR, blocks its activation that suppresses the EGFR‐STAT3 pathway which in turn reduces the promoter activity of Sterol regulatory element binding transcription factor 1 (Srebf1) that is a key regulator of lipogenesis [33]. In a prospective human cohort, circulating IGFBP2 concentrations were lower in individuals with SLD and were inversely associated with BMI, triglycerides, fasting glucose and insulin; importantly, higher baseline IGFBP2 levels were associated with a lower incidence of NAFLD during 3 years of follow‐up [34]. Mechanistic evidence further supports a protective role of IGFBP2 in hepatic lipid metabolism. Zhai et al. identified IGFBP2 as one of the most consistently downregulated genes in human SLD datasets and confirmed reduced hepatic IGFBP2 expression in patients with SLD [33]. These mechanisms highlight the significance of liver‐derived proteins, providing potential insights into diagnosis, prognosis and therapeutic targets.

Direct comparison between MASLD and MetALD showed no significantly differentially expressed proteins and therefore did not provide evidence for a clear proteomic distinction between the groups under the current thresholds (Figure S1). These findings suggest that while MASLD and MetALD share a core metabolic and inflammatory proteomic signature, moderate alcohol exposure may subtly modulate additional pathways relevant to toxin metabolism and adipose tissue dysfunction. However, given the limited MetALD sample size and potential alcohol‐exposure misclassification, these findings should not be interpreted as evidence against MetALD as a distinct disease entity. Larger studies with more precise alcohol‐exposure assessment are needed to determine whether the current thresholds correspond to a reproducible molecular distinction. As alcohol exposure was assessed using self‐reported intake, which may be affected by underreporting and misclassification. Direct alcohol biomarkers such as PEth [4] and EtG [5], as well as CDT [35], were not available in the UKB dataset. Although exploratory PCA analyses based on alcohol consumption categories did not show clear global proteomic separation between MASLD and MetALD, exposure misclassification may have attenuated alcohol‐related differences.

Our findings can also be considered alongside the recent UK Biobank study by Liu et al. [10], which derived a 10‐protein panel (MAMDC4, SSC4D, CEACAM16, CHI3L1, GGT1, C4BPB, CDHR5, OXT, FCAMR and ADAM22) to distinguish ALD from MASLD and then used this panel to subtype MetALD. Two of these proteins, CHI3L1 and OXT, were also significantly upregulated in our MetALD‐versus‐non‐steatotic‐control comparison, while SSC4D was significantly upregulated in our MASLD‐versus‐control comparison. The remaining proteins from the Liu et al. panel did not meet our prespecified MetALD‐versus‐control differential‐abundance criteria. These results are not directly discordant because the Liu et al. proteins were selected for ALD‐versus‐MASLD discrimination rather than for MetALD‐versus‐control differential abundance; in addition, their primary SLD definition used fatty liver index with MRI‐PDFF as a sensitivity analysis, whereas our primary phenotype was MRI‐PDFF‐defined at the proteomics and imaging visit. Together, the studies support molecular heterogeneity within MetALD and suggest that alcohol‐related proteomic signals may depend strongly on the comparator and phenotyping strategy.

### 4.1 Limitations

This study has several limitations. UKB participants are predominantly of European ethnicity, thereby limiting the generalizability of the findings to more ethnically diverse populations. We acknowledge that alcohol consumption in UK Biobank is self‐reported and may be subject to underestimation due to recall and social desirability bias [36]. The voluntary nature of participation in the UK Biobank might also introduce a ‘healthy volunteer effect’, as individuals who choose to participate tend to be healthier than the general population, thus potentially attenuating the observed disease associations [37]. We acknowledge that alcohol exposure based on self‐report is vulnerable to underestimation and misclassification, particularly in population‐based cohorts such as UKB. Direct alcohol metabolites such as PEth and EtG, and established alcohol‐specific biomarkers such as CDT, were not available. Therefore, direct biomarker‐based triangulation could not be performed. The cross‐sectional design inherent in much of the UK Biobank data also precludes drawing causal inferences from the observed associations. These limitations should be considered when interpreting the observed protein associations.

This study highlights the potential of plasma proteomics to improve non‐invasive diagnosis and stratification in SLD. The MASLD‐associated protein signature, including IGFBP1, leptin, FABP1 and GSTA1, may serve as a foundation for future biomarker panels that reflect underlying disease biology. The lack of clear proteomic distinction between MASLD and MetALD, despite marked differences in alcohol intake, did not provide clear proteomic support for the current intake‐based subclassification, although interpretation is limited by the small MetALD sample size.

Our findings suggest that moderate alcohol use may modulate, but did not clearly define, the proteomic phenotype in this cohort. Longitudinal proteomic profiling could also help track disease progression over time, aiding clinical decision‐making. However, further research is required to evaluate the feasibility and clinical relevance of targeting these pathways.

## Author Contributions

The original idea was conceived by C.V.S., with guidance provided by Y.R. N.J. performed the analysis, prepared the main text, figures and tables. T. Seibel derived the alcohol intake measures and generated the disease labels used in the analyses. M.M. performed the external validation. All authors reviewed the results, provided major comments and contributed to the revision of the final manuscript.

## Funding

J.C. is supported by the Mildred‐Scheel‐Postdoktorandenprogramm of the German Cancer Aid (grant #70115730). C.V.S. is supported by a grant from the Interdisciplinary Centre for Clinical Research within the faculty of Medicine at the RWTH Aachen University (PTD 1‐13/IA 532313), the Junior Principal Investigator Fellowship program of RWTH Aachen Excellence strategy and the NRW Rueckkehr Programme of the Ministry of Culture and Science of the German State of North Rhine‐Westphalia. K.M.S. is supported by the Federal Ministry of Education and Research (BMBF) and the Ministry of Culture and Science of the German State of North Rhine‐Westphalia under the Excellence strategy of the federal government and the Laender as well as the NRW Rueckkehr Programme of the Ministry of Culture and Science of the German State of North Rhine‐Westphalia. C.V.S. and K.M.S. are supported by the CRC 1382 project A11 and B09 funded by Deutsche Forschungsgesellschaft (DFG, German Research Foundation) Project‐ID 403224013 SFB 1382. M.M. and Y.R. are supported by the Intramural Research Program of the National Institute of Diabetes and Digestive and Kidney Diseases (NIDDK). T.S. is supported by a grant from the Interdisciplinary Centre for Clinical Research within the faculty of Medicine at the RWTH Aachen University (PTD 1‐13/IA 532313).

## Conflicts of Interest

J.C. has received honoraria from Johnson & Johnson. C.V.S. received a travel grant from Ipsen and Falk in 2025 and consulting honoraria from Pfizer, AstraZeneca, Takeda, Madrigal and AirNA in 2025 and 2026.

## Supporting information


**Figure S1:** Direct comparison of plasma protein abundance between MASLD and MetALD. The volcano plot shows the adjusted disease‐status coefficient from the age‐, sex‐ and BMI‐adjusted protein‐wise linear model on the *x*‐axis and −log10(Bonferroni‐adjusted *p*‐value) on the *y*‐axis (MASLD *n* = 165; MetALD *n* = 46; 1463 proteins tested). No protein met both the Bonferroni‐adjusted significance threshold and the prespecified effect‐size criterion. The annotation summarizes the 80%‐power minimum detectable effect for this comparison (approximately 0.46 log_2_ NPX units; approximately 1.38‐fold).
**Table S1:** Protein‐level missingness among the top MASLD discovery proteins.
**Table S2:** Proteins upregulated in MASLD versus controls after covariate adjustment, Bonferroni correction and effect‐size filtering.
**Table S3:** Proteins downregulated in MASLD versus controls after covariate adjustment, Bonferroni correction and effect‐size filtering.
**Table S4:** Cross‐cohort comparison of the eight UKB discovery proteins measured in the external validation cohort.
**Table S5:** Minimum detectable effect analysis.

## Data Availability

The data that support the findings of this study are available from UK Biobank. Restrictions apply to the availability of these data, which were used under license for this study. Data are available from https://www.ukbiobank.ac.uk/ with the permission of UK Biobank.
