# Supplementary material for: Distinct Plasma Proteomic Signatures Distinguish MASLD From Non‐Steatotic Individuals but Not From MetALD
Source: Liver Int. 2026 Sep 29;46(10):e70873. doi: 10.1111/liv.70873 (PMC13623009; doi:10.1111/liv.70873)
Supplement: Supplementary file 1 [file LIV-46-0-s001.docx]

**Supplementary Material**

**Supplementary Methods**

### *Alcohol Consumption Calculations*

Alcohol intake from all relevant beverage types is converted into grams of pure ethanol per day (g/day) and combined to obtain the participant’s total average daily alcohol consumption [1].

The following assumptions were applied for each beverage type:

Pure ethanol is assumed to have a density of 0.789 g/ml, which is used to convert the volume of pure alcohol into grams of alcohol. General conversion formula used:

drink volume (mL) × alcohol by volume (ABV) × 0.789 g/mL.

- Red wine: Units × (125 mL × 13% ABV × 0.789 g/mL) ÷ 7 or 30.44
- White wine: Units × (125 mL × 11.5% ABV × 0.789 g/mL) ÷ 7 or 30.44
- Beer: Units × (568 mL × 4.5% ABV × 0.789 g/mL) ÷ 7 or 30.44
- Spirits: Units × (25 mL × 40% ABV × 0.789 g/mL) ÷ 7 or 30.44
- Fortified wine: Units × (50 mL × 20% ABV × 0.789 g/mL) ÷ 7 or 30.44
- Other: Units × 8 g ÷ 7 or 30.44

Weekly and monthly consumption: UKB Field 1558 determines the questionnaire branch. Weekly beverage fields are collected for participants who report drinking more often than once or twice per week, whereas monthly fields are collected for participants who drink on special occasions or 1-3 times per month (see UKB Data-Fields 1558, 1608, and 4451). Weekly and monthly values are therefore treated as alternative questionnaire branches rather than duplicate measurements.

Time conversion: One week was defined as 7 days and one month as 365.25/12=30.44 days. Weekly or monthly beverage intake was converted to an average daily intake in g/day.

UKB special codes: negative UKB values representing special responses, such as “Do not know” or “Prefer not to answer”, are converted to missing values “NA” and are not treated as actual consumption amounts. If detailed alcohol-consumption quantities are unavailable, only current drinking status (yes/no) is used as a fallback. Previous drinking status is ignored in this fallback rule.

*Definition of other excluded liver diseases:*

Alcohol-related liver disease (ALD) was defined as hepatic steatosis above the prespecified PDFF threshold together with alcohol exposure above the MetALD range (>50 g/day for women or >60 g/day for men; equivalent to >350 g/week or >420 g/week, respectively) and/or a recorded ICD-10 code beginning with K70.

At-risk MASH was defined as cT1 >875 ms, PDFF above the prespecified steatosis threshold, at least one cardiometabolic risk factor, and alcohol consumption below the ALD threshold.

Cryptogenic liver disease was defined operationally as PDFF above the prespecified steatosis threshold in the absence of cardiometabolic risk factors. Other potential causes of liver disease were not comprehensively excluded in this definition.

Cirrhosis was defined from previously recorded diagnoses using the prespecified ICD code list, as follows: K746: Other and unspecified cirrhosis of liver, K717: Toxic liver disease with fibrosis and cirrhosis of liver, K766: Portal hypertension

Other liver diseases were defined from previously recorded diagnoses using the prespecified ICD code list for other liver diseases, listed below: B18: Chronic Viral Hepatitis, K73: Chronic Hepatitis, K713: Toxic liver disease with chronic persistent hepatitis, K715: Toxic liver disease with chronic active hepatitis, K721: Chronic hepatic failure, K743: Primary biliary cirrhosis (PBC), K754: Autoimmune hepatitis

### *Gene concept network*

The gene-concept network was represented as a bipartite graph G=(U, V, E), where U denotes genes and V denotes significantly enriched biological pathways or concepts. An edge e=(u,v) was drawn when gene u was annotated to concept v in the enrichment database.

**References**

[1]	Moore SC, Orpen B, Smith J, et al. Alcohol affordability: implications for alcohol price policies. A cross-sectional analysis in middle and older adults from UK Biobank. J Public Health (Oxf) 2022;44: e192–202. https://doi.org/10.1093/pubmed/fdab095.

### **Supplementary Table 1. Protein-level missingness among the top MASLD discovery proteins.**

| **Protein** **Name** | **Direction** | **Model** **n** | **Total missing** **n (%)** |
| --- | --- | --- | --- |
| IGFBP2 | downregulated | 905 | 0 (0.00%) |
| GSTA1 | upregulated | 892 | 13 (1.43%) |
| CES1 | upregulated | 905 | 0 (0.00%) |
| ADH4 | upregulated | 883 | 22 (2.43%) |
| GUSB | upregulated | 898 | 7 (0.77%) |
| ACY1 | upregulated | 897 | 8 (0.88%) |
| KRT18 | upregulated | 881 | 24 (2.65%) |
| RBP5 | upregulated | 881 | 24 (2.65%) |
| SSC4D | upregulated | 897 | 8 (0.88%) |
| GSTA3 | upregulated | 869 | 36 (3.97%) |
| LEP | upregulated | 897 | 8 (0.88%) |
| CDHR2 | upregulated | 881 | 24 (2.65%) |
| IGFBP1 | downregulated | 905 | 0 (0.00%) |
| FGF21 | upregulated | 890 | 15 (1.66%) |
| OXT | upregulated | 901 | <5 (<0.5%) |
| CA5A | upregulated | 883 | 22 (2.43%) |
| FABP1 | upregulated | 894 | 11 (1.21%) |
| HAO1 | upregulated | 890 | 15 (1.66%) |
| GH1 | downregulated | 897 | 8 (0.88%) |
| AIFM1 | upregulated | 881 | 24 (2.65%) |

*Sample sizes reflect participants with complete data for disease status, protein abundance, and covariates included in the adjusted models. One participant was removed because of missing covariate information.*

**Supplementary Table 2. Proteins upregulated in MASLD versus controls after covariate adjustment, Bonferroni correction, and effect-size filtering.**

| **Protein Name** | **Adjusted log_2_ fold change** **(Adjusted beta)** | **P-value adjusted Bonferroni** |
| --- | --- | --- |
| GSTA1 | 1.00 | 14.122e-32 |
| CES1 | 0.88 | 1.0945e-31 |
| ADH4 | 1.01 | 8.546e-31 |
| GUSB | 0.65 | 6.547e-26 |
| ACY1 | 0.59 | 4.802e-22 |
| KRT18 | 0.74 | 4.936e-22 |
| RBP5 | 0.52 | 3.252e-20 |
| SSC4D | 1.05 | 3.724e-19 |
| GSTA3 | 0.73 | 1.317e-18 |
| LEP | 0.72 | 1.489e-16 |
| CDHR2 | 0.60 | 3.411e-15 |
| FGF21 | 0.87 | 2.355e-12 |
| OXT | 0.87 | 4.428e-07 |
| CA5A | 0.56 | 1.126e-06 |
| FABP1 | 0.52 | 4.004e-06 |
| HAO1 | 0.69 | 7.819e-05 |
| AIFM1 | 0.51 | 1.444e-02 |

**Supplementary Table 3. Proteins downregulated in MASLD versus controls after covariate adjustment, Bonferroni correction, and effect-size filtering.**

| **Protein** **name** | **Adjusted log_2_ fold change** **(Adjusted beta)** | **P-value adjusted Bonferroni** |
| --- | --- | --- |
| IGFBP2 | -0.76 | 9.133e-36 |
| IGFBP1 | -1.06 | 1.063e-14 |
| GH1 | -0.88 | 2.736e-03 |

**Supplementary Table 4.** Cross-cohort comparison of the eight UKB discovery proteins measured in the external validation cohort.

| **Protein name** | **Adjusted log_2_ fold change** **(Adjusted beta)** **(UKB) between MASLD and Controls** | **P-value adjusted Bonferroni** **(UKB)** | **External effect: geometric FC; Hedges g (95% CI)** | **External validation P-value** | **External validation BH-FDR q-value** |
| --- | --- | --- | --- | --- | --- |
| ACY1 | 0.59 | 4.802e-22 | 3.95; 1.85 (0.95 to 2.74) | 1.287e-06 | **1.030e-05** |
| KRT18 | 0.74 | 4.936e-22 | 0.96; -0.12 (-0.92 to 0.68) | 0.601 | 0.772 |
| GSTA3 | 0.73 | 1.317e-18 | 0.74; -0.50 (-1.30 to 0.31) | 0.676 | 0.772 |
| LEP | 0.72 | 1.489e-16 | 5.05; 2.06 (1.14 to 2.98) | 0.0018 | **0.0071** |
| FABP1 | 0.52 | 4.004e-06 | 2.34; 1.29 (0.44 to 2.14) | 0.0055 | **0.0148** |
| IGFBP2 | -0.76 | 9.133e-36 | 0.71; -1.10 (-1.93 to -0.26) | 0.0688 | 0.110 |
| IGFBP1 | -1.06 | 1.063e-14 | 0.47; -1.21 (-2.06 to -0.37) | 0.0182 | **0.0364** |
| GH1 | -0.88 | 2.736e-03 | 1.01; 0.01 (-0.79 to 0.81) | 0.862 | 0.862 |

*The UKB columns report the adjusted disease-status coefficient (beta) from the MASLD-versus-control model and the corresponding Bonferroni-adjusted p-value. External effect sizes are reported as the geometric fold change and Hedges' g with 95% confidence interval on log2-transformed values. Nominal external p-values are from two-sided exact Wilcoxon rank-sum (Mann-Whitney U) tests. The eight overlapping proteins constituted a single external-validation multiple-testing family; Benjamini-Hochberg FDR correction was applied jointly across all eight nominal p-values (m=8), irrespective of direction of regulation. q≤0.05 was considered significant.*

**Supplementary Table 5. Minimum detectable effect analysis**

| **Comparison** | **Group 1** **n** | **Group 2** **n** | **Proteins** **tested** | **Bonferroni** **alpha** | **Power** | **Min detectable** **Cohen's d** | **Median protein** **SD*** | **Approx. min** **log_2_ NPX difference** | **Approx. min** **fold-change** |
| --- | --- | --- | --- | --- | --- | --- | --- | --- | --- |
| MASLD vs Control | 165 | 741 | 1463 | 3.418e-05 | 0.80 | 0.431 | 0.566 | 0.244 | 1.184 |
| MetALD vs Control | 46 | 741 | 1463 | 3.418e-05 | 0.80 | 0.762 | 0.560 | 0.427 | 1.344 |
| MASLD vs MetALD | 165 | 46 | 1463 | 3.412e-05 | 0.80 | 0.849 | 0.542 | 0.460 | 1.38 |

** Median protein SD was used to convert the minimum detectable Cohen's d to an approximate log2 NPX difference and fold change for all three comparisons. These conversions are descriptive aids and do not represent observed effect sizes.*

### **Supplementary Figures**

**Supplementary Figure 1: Direct comparison of plasma protein abundance between MASLD and MetALD.** The volcano plot shows the adjusted disease-status coefficient from the age-, sex-, and BMI-adjusted protein-wise linear model on the x-axis and -log10(Bonferroni-adjusted p-value) on the y-axis (MASLD n=165; MetALD n=46; 1,463 proteins tested). No protein met both the Bonferroni-adjusted significance threshold and the prespecified effect-size criterion. The annotation summarizes the 80%-power minimum detectable effect for this comparison (approximately 0.46 log_2_ NPX units; approximately 1.38-fold).
